# The complete mitochondrial genomes of *Paradiplozoon yarkandense* and *Paradiplozoon homoion* confirm that Diplozoidae evolve at an elevated rate

**DOI:** 10.1186/s13071-022-05275-x

**Published:** 2022-04-27

**Authors:** Cui-Lan Hao, Kadirden Arken, Munira Kadir, Wen-Run Zhang, Meng-Jie Rong, Nian-Wen Wei, Yan-Jun Liu, Cheng Yue

**Affiliations:** College of Veterinary Medicine, Xinjiang Agricultural University, Urumqi, 830052 Xinjiang China

**Keywords:** Polyopisthocotylea, Monogenea, Indodiplozoon, Diplozoon, Base composition, Phylogeny, Evolution, Gene overlap, Mitogenome

## Abstract

**Background:**

Diplozoidae are monogenean (Monogenea: Polyopisthocotylea) fish parasites characterised by a unique life history: two larvae permanently fuse into an X-shaped “Siamese” organism. Taxonomy and phylogeny of Diplozoidae and Polyopisthocotylea remain unresolved due to the unavailability of molecular markers with sufficiently high resolution. Mitogenomes may be a suitable candidate, but there are currently only 12 available for the Polyopisthocotylea (three for Diplozoidae). The only available study of diplozoid mitogenomes found unique base composition patterns and elevated evolution rates in comparison with other Monogenean mitogenomes.

**Methods:**

To further explore their evolution and generate molecular data for evolutionary studies, we sequenced the complete mitogenomes of two Diplozoidae species, *Paradiplozoon homoion* and *Paradiplozoon yarkandense*, and conducted a number of comparative mitogenomic analyses with other polyopisthocotyleans.

**Results:**

We found further evidence that mitogenomes of Diplozoidae evolve at a unique, elevated rate, which was reflected in their exceptionally long branches, large sizes, unique base composition, skews, and very low gene sequence similarity levels between the two newly sequenced species. They also exhibited remarkably large overlaps between some genes. Phylogenetic analysis of Polyopisthocotylea resolved all major taxa as monophyletic, and Mazocraeidea was split into two major clades: (Diplozoidae) + (all four remaining families: Diclidophoridae, Chauhaneidae, Mazocraeidae and Microcotylidae). It also provided further confirmation that the genus *Paradiplozoon* is paraphyletic and requires a taxonomic revision, so the two species may have to be renamed *Indodiplozoon homoion* and *Diplozoon yarkandense* comb. nov.

**Conclusions:**

Although our findings indicate that mitogenomes may be a promising tool for resolving the phylogeny of Polyopisthocotylea, elevated evolutionary rates of Diplozoidae may cause phylogenetic artefacts, so future studies should pay caution to this problem. Furthermore, as the reason for their elevated evolution remains unknown, Diplozoidae are a remarkably interesting lineage for other types of evolutionary mitogenomic studies.

**Graphical Abstract:**

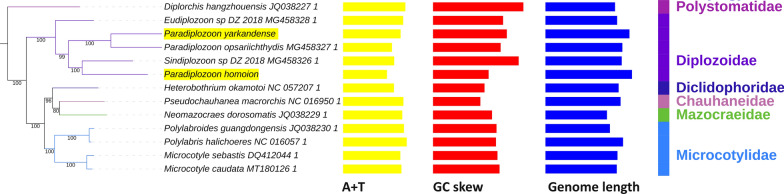

**Supplementary Information:**

The online version contains supplementary material available at 10.1186/s13071-022-05275-x.

## Background

Flatworms are a phylum (Platyhelminthes) of largely parasitic animals of high importance for medicine, as they cause diseases in a variety of host animal groups, including domestic animals and humans. They also exhibit a broad range of parasitic strategies, including some lineages exhibiting remarkably high host-specificity [1–3]. As Platyhelminthes also comprises free-living lineages, these factors make Platyhelminthes a good phylogenetic system for studying the evolution of parasitism. The predominantly parasitic three classes of flatworms are classified together as the superclass Neodermata: Monogenea (primarily ectoparasitic), Trematoda (endoparasitic flukes), and Cestoda (endoparasitic tapeworms). Whereas the monophyly of Neodermata is relatively robust, the relationships among the above three main clades, as well their monophyly, remain debated [2–6]. Notably, there is evidence that Monogenea, which comprises a major part of the obligate parasitic flatworm diversity, might be paraphyletic, and split into two independent radiations (subclasses): *Polyopisthocotylea* and *Monopisthocotylea* [6, 7]. Mitogenomic nucleotide sequences and gene order rearrangements indicate that *Polyopisthocotylea* may even be a sister group to all other Neodermata [6, 8]. Another intriguing feature of this subclass is that it also exhibits mitogenomes with rearranged gene orders; this is uncommon within the Neodermata, which mostly exhibit a highly conserved architecture [6].

Along with the family Octomacridae, Diplozoidae are the only polyopisthocotyleans primarily parasitizing freshwater teleosts [9]. They are predominantly ectoparasites, commonly found on the gills of cyprinid and characid fishes, and they have a direct life-cycle (no intermediate host), which is typical for monogeneans. However, Diplozoidae are also characterised by a unique life history: two larvae (diporpae) permanently fuse into a pair, morphing into an X-shaped “Siamese” organism [9–11]. They are also important for aquaculture because they cause notable damage to the gill tissue of their hosts, which may cause secondary infections and mortality [11].

Taxonomy and phylogeny of diplozoids are mainly based on morphology (central hooks, clamps and spermatozoid ultrastructure) and host fishes, but reliance on these parameters often causes taxonomic and phylogenetic artefacts [11–13]. Therefore, the availability of molecular markers is a prerequisite for the reliable identification and phylogenetic and taxonomic studies of diplozoid parasites. Previous phylogenetic studies mostly relied on the *28S* and ITS-2 rDNA (second internal transcribed spacer of ribosomal DNA) sequences, which often produce incongruent results between different datasets and methods [11, 13–16]. This lack of reliable phylogenetic marker results in multiple unresolved phylogenetic and taxonomic questions within the Diplozoidae and Monogenea [6, 13]. Mitochondrial genomes (mitogenomes) can provide a phylogenetic resolution superior to the traditionally used single-gene markers, so they are becoming an increasingly popular tool in evolutionary, population genetic, taxonomic, phylogenetic and diagnostic studies of Platyhelminthes [7, 11, 17, 18]. The only previous study that applied mitochondrial phylogenomics to Diplozoidae produced some novel family-level relationships, which indicates that the usefulness of mitogenomes for inferring the phylogeny of Polyopisthocotylea should be further explored [11, 13].

In most animal lineages, mitogenomes are circular molecules, ranging 13 ~ 16 kb in size, that encode 37 genes—13 protein-coding genes (PCGs), two ribosomal RNA genes, and 22 transfer RNA (tRNA) genes—but comparative mitogenomic architecture analyses (e.g., gene arrangement, base composition skews, etc.) often reveal lineages that exhibit intriguing patterns of mitogenomic evolution [19–22]. The only published study of Diplozoidae mitogenomes found unique base composition patterns and elevated evolution rates in comparison with other Monogenean mitogenomes [11]. As there are currently (Dec. 2021) only 12 sequenced and annotated complete mitogenomes for the Polyopisthocotylea, this scarcity of data hampers progress in the understanding of mitogenomic architecture evolution and phylogeny of Polyopisthocotylea and Monogenea. To address this dearth of data and further explore the unique base composition of diplozoid mitogenomes, we sequenced complete mitogenomes of two Diplozoidae species: *Paradiplozoon homoion* (Bychowsky & Nagibina, 1959) and a recently described [23] new species *Paradiplozoon yarkandense* (Arken et al. [23]).

## Methods

*Paradiplozoon yarkandense* was collected from the host *Diptychus maculatus* in the Taxkorgan river (a tributary of the Yarkand River) (37° 41′ 14″ N; 75° 18′ 9″ E), Xinjiang, China. *Paradiplozoon homoion* was collected from the host *Leuciscus baicalensis* in the Kelan River (a tributary of the Irtysh River) (47° 42′ 40″ N, 88° 13′ 25″ E). Xinjiang, China. Both species were morphologically identified according to our previous publications [23, 24] (Additional file 1: Text S1 and Figs. S1 to S5). DNA extraction, mitogenome amplification and sequencing, and sequence annotation and analyses were conducted exactly as described before [11]. The methodology only differed in selecting different reference mitogenomes: *Polylabris halichoeres* [25] and the three available Diplozoidae species [11] and different primers (Additional file 1: Tables S1 and S2). tRNAs were first identified using ARWEN [26] and MITOS [27] programs, and then R2DT [28] was further used to predict and visualise the secondary structure of selected tRNAs. ORF-Finder [29] was further used to search for genes resembling atp8 in large non-coding regions of both mitogenomes. Phylogenetic analysis was conducted on a dataset comprising all available Polyopisthocotylea mitogenomes (Additional file 2: Dataset S1). Following the evidence that using multiple outgroups produces better results than using a single outgroup [30], we used two different lineages as outgroups, two mitogenomes from the relatively closely related neodermatan clade, Monopisthocotylea: *Gyrodactylus salaris* (Gyrodactyloidea) [31] and *Dactylogyrus lamellatus* (Dactylogyridea) [32]; and two mitogenomes from Rhabditophora, belonging to the basal radiation of largely free-living flatworms [5]: *Bipalium kewense* [33] and *Obrimoposthia wandeli* [34]. PhyloSuite [35] was used to standardise annotation, extract data, and conduct phylogenetic analysis in the Flowchart mode using nucleotide sequences of 12 concatenated (but partitioned) PCGs with the help of several plug-in programs. Genes were aligned using the codon mode and the accurate G-INS-i strategy in MAFFT [36], concatenated using PhyloSuite, best-suited evolutionary models for partitions inferred using ModelFinder [37], and phylogeny was reconstructed using IQ-TREE [38] with 10,000 ultrafast bootstraps [39]. iTOL [40] was used to visualise the phylogeny and architecture using files generated by PhyloSuite. OGDRAW was used to create to-scale circular maps of the mitogenomic architecture [41].

## Results

### Phylogeny and identity

**Fig. 1:**
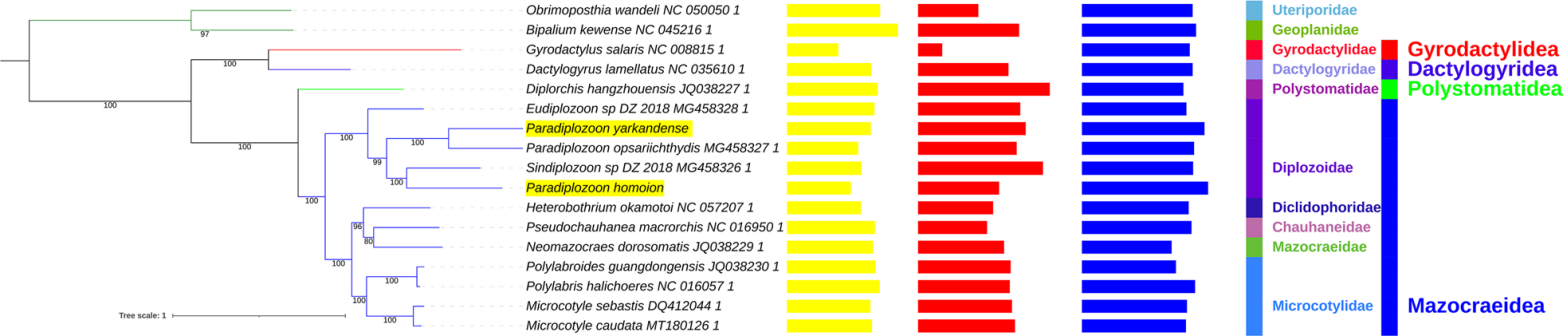
The mitogenomic phylogeny of Polyopisthocotylea. Species names are followed by the GenBank accession number, statistical support is shown next to branches, and the tree scale is included in the figure. Yellow bars correspond to the A+T composition of entire mitogenomes, red bars correspond to the GC skew, and blue bars to the mitogenome length. Taxonomic identity is shown to the right: family and order (apart from Rhabditophora)

The ITS-2 sequence of the putative *P. yarkandense* exhibited 100% identity to a number of orthologous *P. yarkandense* sequences published in the original description study [23], whereas that of *P. homoion* exhibited a 100% identity to a *P. homoion* ITS-2 sequence sequenced by Benovics et al. [42] (Additional file 1: Figs. S5 and S6). The phylogenetic analysis resolved all major taxa, including the Diplozoidae, as monophyletic (Fig. 1). Statistical support for clades was very high (mostly over 90%). Mazocraeidea was split into two major clades; one comprising only Diplozoidae and the other containing all four remaining families included in the dataset (Diclidophoridae, Chauhaneidae, Mazocraeidae, and Microcotylidae). Within the Diplozoidae, however, the genus *Paradiplozoon* was paraphyletic: *P. yarkandense* formed a sister lineage with *Paradiplozoon opsariichthydis*, whereas *P. homoion* formed a sister lineage with *Sindiplozoon* sp. *Eudiplozoon* sp. was the sister group to the remaining four species.

### Mitochondrial architecture and size

**Fig. 2:**
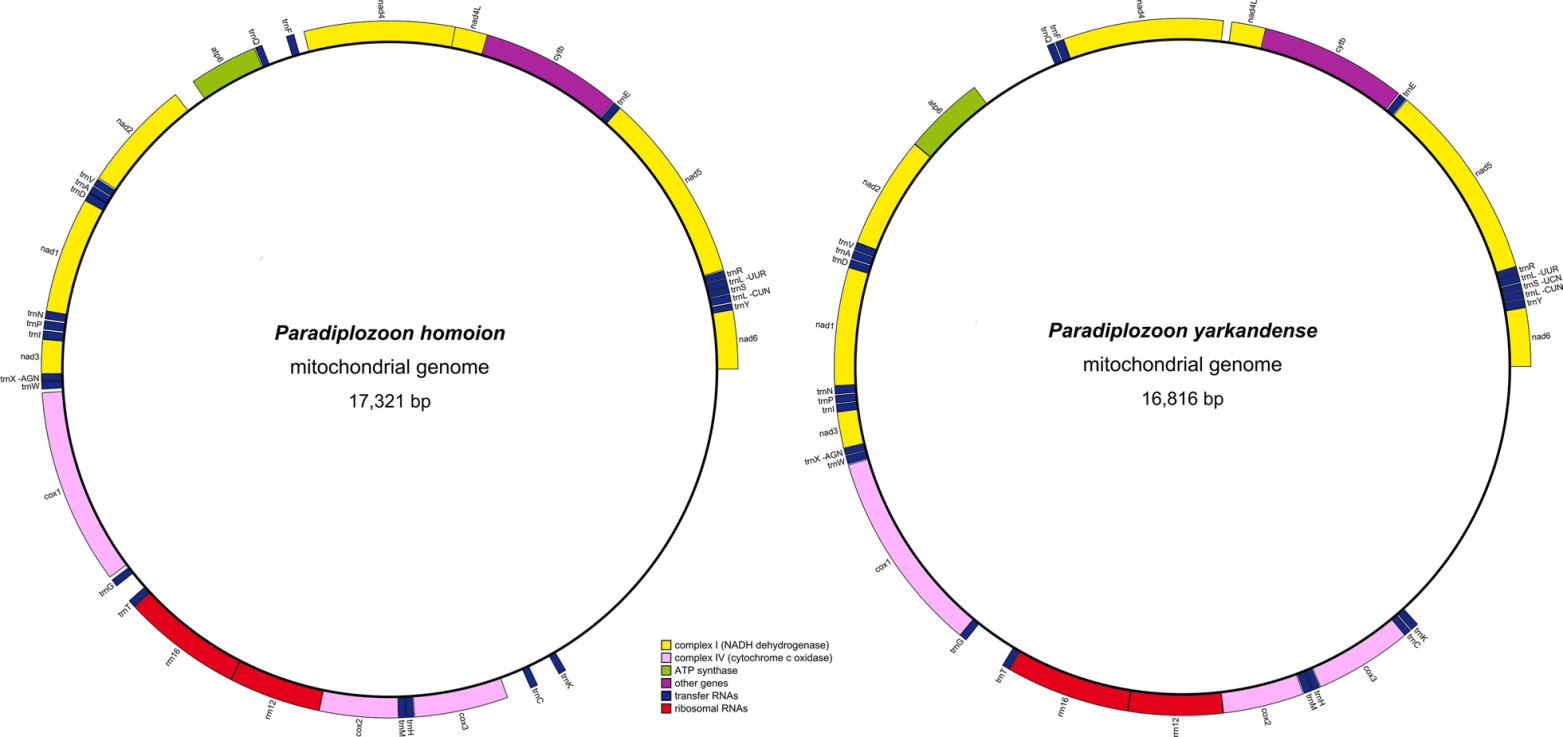
To-scale circular architectural maps of mitogenomes of *P. homoion* and *P. yarkandense.* The transcription direction is clockwise

**Fig. 3:**
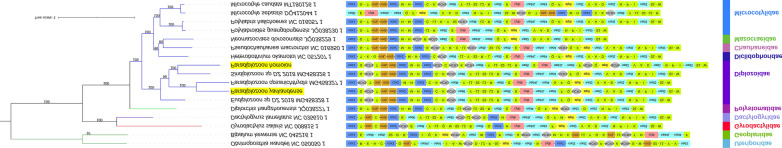
Gene orders in Polyopisthocotylea. Species names are followed by GenBank accession numbers. The two newly sequenced species are shaded yellow. Family-level taxonomic identity is shown to the right. Partial mitogenomes are labelled with the letter P

All genes are encoded on the same strand and the *atp8* gene is missing from both newly sequenced mitogenomes (Fig. 2; Table 1)*.* Both exhibit a gene order identical to that of *Eudiplozoon* sp. and *Sindiplozoon* sp. The only difference they exhibited was in the distribution of non-coding regions larger than 100 base pairs (bp) (Figs. 2 and 3), which is indicative of the fast evolution of non-coding sections. Both mitogenomes were the largest among the sequenced polyopisthocotylean mitogenomes: *P. homoion* was the largest with 17,321 bp and *P. yarkandense* was the second largest, with 16,816 bp (Fig. 1, Additional file 2: Dataset S1). Moreover, all other available mitogenomes were much smaller: 12,290 to 15,527 bp (some of the smallest mitogenomes were incomplete, so the lower end of the range is almost certainly a sequencing or assembly artefact). Aside from the outlier of *P. halichoeres* (Microcotylidae; 15,527 bp), the top five largest mitogenomes belonged to Diplozoidae. Aside from *Eudiplozoon* sp. (14,334 bp), all other Diplozoidae mitogenomes were larger than 15 kbp.

**Table 1:** The comparative table of mitogenomic architectures of *P. yarkandense* (left) and *P. homoion* (right)

Gene	Position		Size	IGR^a^	Codon	
	From	To			Start	Stop
*nad6*	1/1	460/477	460/477		ATG/ATG	T–/TAG
*trnY*	461/471	525/527	65/57	0/−7		
*trnL1*	527/535	593/601	67/67	1/7		
*trnS2*	595/604	653/661	59/58	1/2		
*trnL2*	657/662	722/727	66/66	3/0		
*trnR*	723/729	786/794	64/66	0/1		
*nad5*	780/798	2336/2339	1557/1542	−7/3	ATT/GTG	TAG/TAA
*trnE*	2341/2340	2404/2404	64/65	4/0		
*cytb*	2412/2408	3566/3559	1155/1152	7/3	ATT/ATG	TAG/TAA
*nad4L*	3557/3544	3826/3810	270/267	−10/−16	GTG/TTG	TAA/TAG
*nad4*	3886/3762	5139/5018	1254/1257	59/−49	ATT/TTG	TAG/TAG
*trnF*	5140/5097	5206/5163	67/67	0/78		
*trnQ*	5213/5365	5274/5424	62/60	6/0		
NCR	5275/5164	5915/5364	641/201			
*atp6*	5916/5430	6551/5985	636/556	0/5	ATG/ATG	TAG/T–
*nad2*	6551/6154	7432/7059	882/906	−1/0	GTG/GTG	TAG/TAA
*trnV*	7431/7067	7494/7130	64/64	−2/7		
*trnA*	7497/7135	7562/7197	66/63	2/4		
*trnD*	7568/7197	7632/7263	65/67	5/−1		
*nad1*	7633/7264	8554/8197	922/934		TTG/TTG	T–/T–
*trnN*	8555/8198	8619/8261	65/64			
*trnP*	8625/8271	8689/8343	65/73	5/9		
*trnI*	8693/8352	8759/8420	67/69	3/8		
*nad3*	8741/8421	9043/8697	303/277	−19/0	ATG/TTG	TAA/T–
*trnS1*	9035/8698	9096/8755	62/58	−9/0		
*trnW*	9099/8756	9166/8819	68/64	2/0		
*cox1*	9170/8844	10,772/10422	1603/1579	3/24	GTG/ATT	T–/T–
*trnG*	10,755/10447	10,819/10511	65/65	−18/24		
NCR_2	10,820/5986	11,156/6153	337/168			
*trnT*	11,157/10660	11,217/10723	61/64			
*rrnL*	11,218/10724	12,186/11685	969/962			
*rrnS*	12,187/11686	12,926/12426	740/741			
*cox2*	12,927/12427	13,562/13071	636/645		ATG/GTG	TAG/TAA
*trnM*	13,566/13057	13,634/13121	69/65	3/−15		
*trnH*	13,635/13122	13,698/13184	64/63			
*cox3*	13,701/13187	14,471/13945	771/759	2/2	ATG/ATG	TAG/TAA
*trnC*	14,462/14138	14,522/14201	61/64	−10/0		
*trnK*	14,528/14389	14,596/14453	69/65	5/0		
NCR_3	14,597/10512	16,816/10659	2220/148			
Overlaps:	9/5	Gaps:	15/14			

^a^Intergenic region (negative values indicate overlaps)

### Base composition and skews

**Fig. 4:**
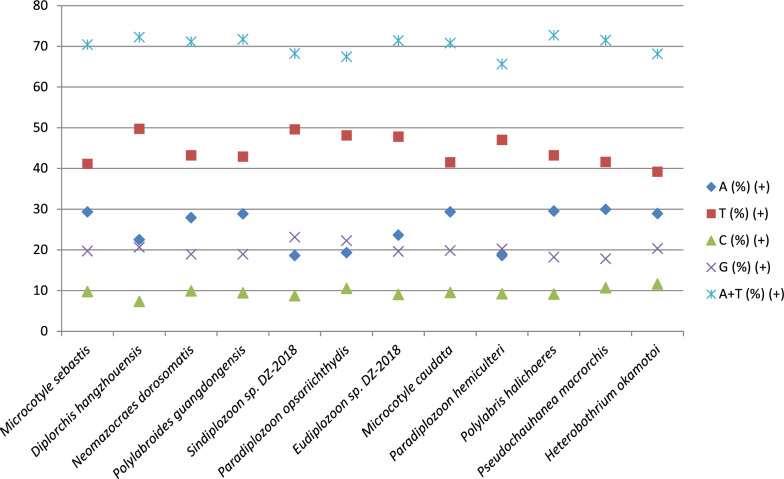
The base composition of mitogenomes of Polyopisthocotylea

The A+T-base content was high in the polyopisthocotylid mitogenomes: 65.6–72.7% (Fig. 1, Fig. 4, Additional file 2: Dataset S1). However, Diplozoidae exhibited the three lowest values in the dataset. This was particularly strongly pronounced in the *P. homoion* mitogenome, which shared the lowest AT content of 65.6% with *Paradiplozoon hemiculteri*. The elevated AT content in the dataset was largely driven by the high T-base content (39.2–50.7%). Somewhat surprisingly, the six Diplozoidae were among the top seven taxa with the highest T-content (45.2–50.7%), along with the Polystomatidae species, *Diplorchis hangzhouensis* (49.7%). Therefore, the reduced AT content in Diplozoidae was driven solely by the strongly reduced A-content (18.6–20.4%) in all Diplozoidae aside from *Eudiplozoon* sp. (23.6%) in comparison with other polyopisthocotylids (22.5–29.9%). The C-content was strongly reduced in all species (7.3–12.1%), but Diplozoidae were not consistently different from the rest of the dataset; the only minor exception was the newly sequenced *P. homoion* with the highest C-content in the dataset (12.1%). In terms of the G-content, Diplozoidae on average exhibited comparatively reduced values (five out of seven lowest values).

**Fig. 5:**
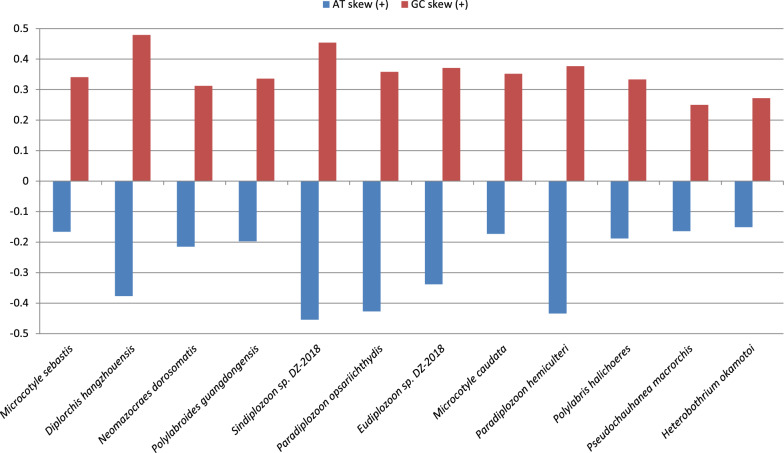
AT and GC skews in mitogenomes of Polyopisthocotylea

The unusual base composition was also reflected in base composition skews (Fig. 1, Fig. 5, Additional file 2: Dataset S1). Diplozoidae had much higher AT skews (−0.34 to −0.45) than the rest of the dataset (−0.15 to −0.22). The only other species that had skews comparable to Diplozoidae was *D. hangzhouensis* (−0.38). As regards GC skews, a majority of Diplozoidae grouped in the upper end of the range (0.358 to 0.454), but the highest value was exhibited by *D. hangzhouensis* (0.479), and *P. homoion* exhibited a low value of 0.294 (the overall range: 0.25 to 0.48).

### Gene comparison

**Fig. 6:**
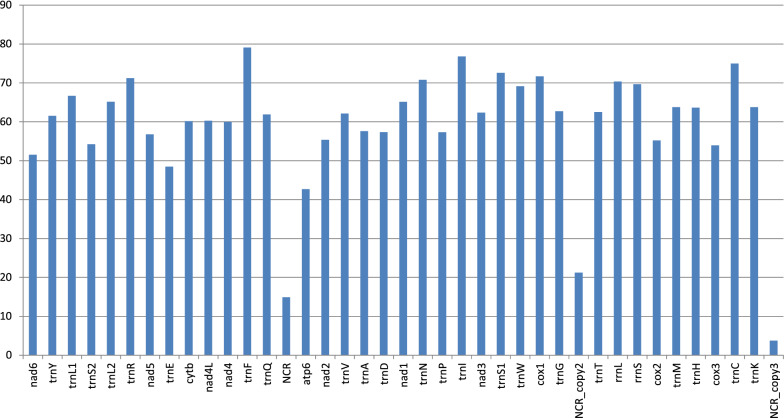
Gene identity (%) values between the genes of *P. yarkandense* and *P. homoion*

For nominally congeneric species, the two newly sequenced *Paradiplozoon* species exhibited remarkably low identity values between genes (Fig. 6). Only three tRNA genes exhibited similarity values larger than 75%: *trnF* (79), *trnI* (77), and *trnC* (75). The two species also exhibited surprisingly divergent start and stop codons (Table 1). Otherwise, start/stop codons were standard for polyopisthocotyleans (Additional file 2: Dataset S2).

This rapid sequence evolution was reflected in a high number of insertions, deletions, and in general poorly aligned gene segments within the Polyopisthocotylea dataset. For example, *nad6* exhibited a 3′ elongation in *P. homoion*, which caused a 7-bp overlap with the downstream *trnY* (Table 1, Additional file 1: Fig. S8). *Nad5* and *nad3* were highly divergent among the available species, with few universally conserved sites, and 5′ and 3′ ends exhibiting many insertions and deletions. *Nad1*, *nad2* and *nad4* were relatively conserved, aside from their highly divergent 5′ ends. Maybe it should be noted that *nad2* in *D. hangzhouensis* had a 3′ extension unmatched in the dataset, so we hypothesise that this is most probably an artefact. Somewhat surprisingly, *nad4L* was relatively conserved within the dataset. As regards *cytb*, 5′ and 3′ ends were highly divergent in the dataset, but the rest was conserved. *cox3* was relatively poorly conserved, with insertions and deletions throughout the sequences, but also with some universally conserved sites. Start codons for this gene were perfectly conserved among all species, but the two newly sequenced species exhibited a 3′ elongation of 6 to 10 bases. *Cox2* was more conserved, with fewer insertions and deletions. In *P. homoion*, *cox2* exhibited a 3′ end elongation of 12 bases. This occurred due to a loss of the T base, which changed the stop codon TAG into –AG and caused a frameshift mutation. As a consequence, there may exist a 15 bp overlap with the downstream *trM* (discussed above). We sequenced this segment twice to confirm this, and found no indications of an annotation artefact. The central part of *cox1* was highly conserved, higher than any other gene, but both 5′ and 3′ ends were remarkably divergent, at least for this usually highly conserved gene [43]. *Paradiplozoon homoion* exhibited an elongation of 15 bp at the 5′ end as compared with *P. yarkandense* and several other species, but these were not the only species that exhibited a 5′ elongation. *Paradiplozoon homoion* exhibited a slightly truncated 3′ end in comparison with most other species (but the 3′ end was not conserved among species so there is no reliable benchmark to compare it with), whereas *P. yarkandense* exhibited a slightly elongated 3′ end. As 3′ was highly divergent in the dataset, we have no reason to suspect sequencing or annotation artefacts. *Atp6* was generally very poorly conserved, with only a few globally conserved sites and highly divergent 3′ end. Py exhibited a large insertion, which resulted in a slightly increased size in comparison with available orthologues (636 bp; compared with 556 in *P. homoion*).

### Gene overlaps

Locations and sizes of intergenic regions and gene overlaps were also remarkably divergent for nominal congenerics (Table 1). Furthermore, several overlaps were very large. Accordingly, in the two studied species, most gene overlaps involved a tRNA gene, but we also identified some overlaps between two PCGs. A relatively large putative overlap between *cox1* and *nad4L* was conserved in both species: *P. yarkandense* = 10 bp, *P. homoion* = 16 bp (Table 1). There was also a putative remarkably large overlap of 49 bases between *nad4* and *nad4L* genes in *P. homoion*. Equally surprisingly, these two genes had 59 bases of intergenic space in *P. yarkandense*. Although *nad4L* exhibited only a handful of universally conserved amino acids in the alignment of polyopisthocotylean orthologues (Additional file 1: Fig. S9), the *P. yarkandense* and *P. homoion* orthologues exhibited the identity of 60% (Fig. 6), which was an average similarity value for PCGs.

**Fig. 7:**
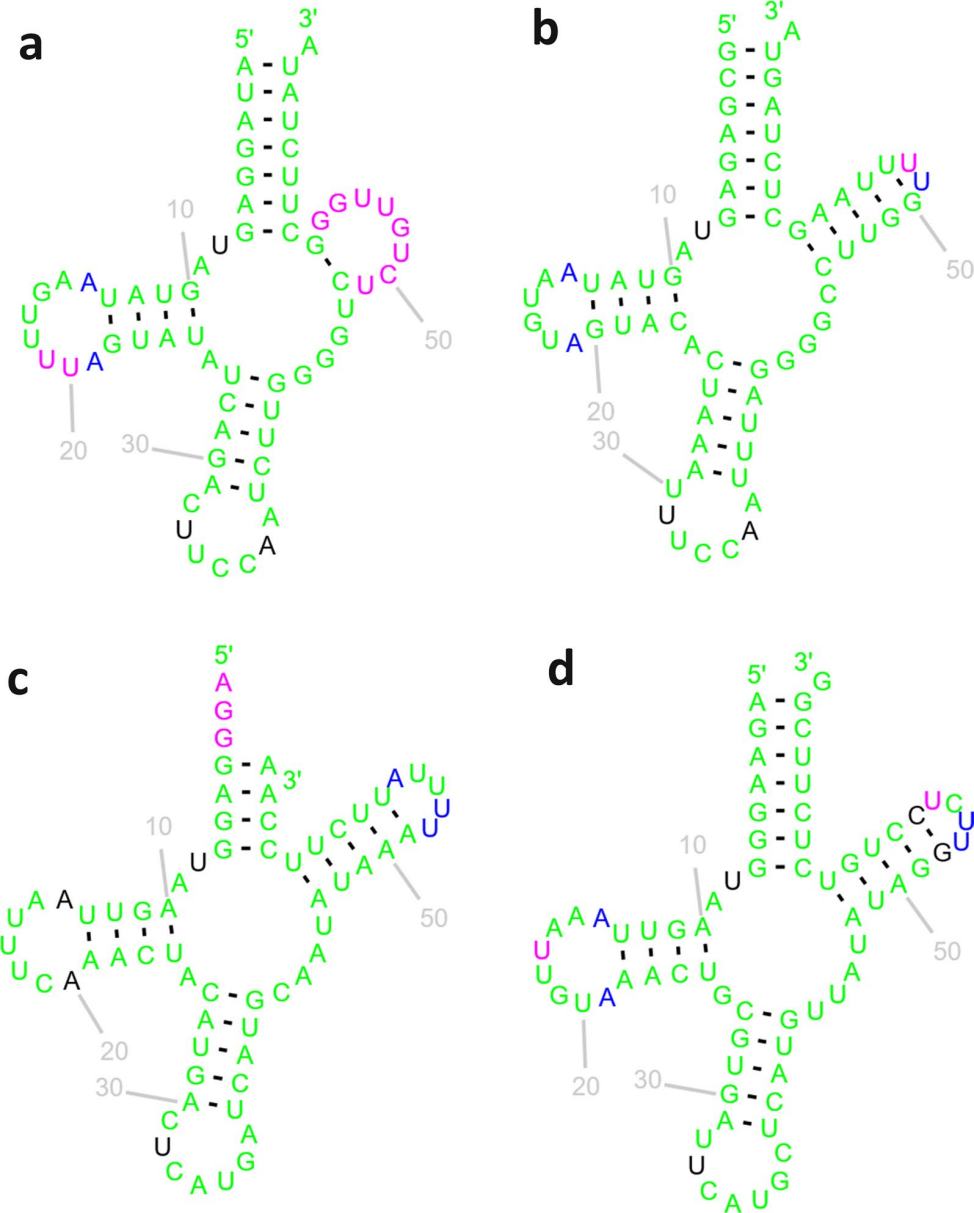
Secondary structures of tRNA genes. **a** *P. yarkandense* trnG. **b** *P. homoion* trnG. **c** *P. homoion* trnM. (**d**) *P. yarkandense* trnM

Other overlaps involved tRNA genes. In *P. yarkandense*, *trnI* exhibited a large overlap of 19 bases with the downstream *nad3*. The alignment of *trnI* genes of Polyopisthocotylea indicated that only the 3′ was not highly conserved, and the *P. yarkandense* orthologue was remarkably similar to other genes (Additional file 1: Fig. S10), as additionally indicated by some of the highest identity levels between the two newly sequenced species of almost 78% (Fig. 6). Notably, this gene was annotated by MITOS, whereas ARWEN and R2DT failed to fold it into a cloverleaf structure. The *trnI* of *P. homoion* was successfully folded into a standard structure (Additional file 1: Fig. S11). It is unclear whether this is an indication of an algorithm artefact, posttranslational editing [44], or non-functionality. *trnG* of *P. yarkandense* overlapped by 18 bases with the upstream *cox1*. This gene was less conserved than *trnI* (Additional file 1: Fig. S12), but it was successfully recognised as a tRNA by all three algorithms employed. It could be folded into a cloverleaf structure, but its T-arm appeared rather crippled (Fig. 7a) as compared with the *P. homoion* orthologue (Fig. 7b) and other polyopisthocotylean orthologues [11]. In *P. homoion*, *trnM* overlapped by 15 bases with the upstream *cox2*. Its 3′ end was rather poorly conserved among the orthologues (Additional file 1: Fig. S13). Structurally, it was successfully folded into a perfectly standard cloverleaf structure, apart from its 3′ end (acceptor stem), which was truncated (Fig. 7c and d).

## Discussion

Phylogenetic analysis of Polyopisthocotylea resolved all major taxa as monophyletic, and Mazocraeidea was split into two major clades: (Diplozoidae) + (all four remaining families: Diclidophoridae, Chauhaneidae, Mazocraeidae and Microcotylidae). It also provided further confirmation that the genus *Paradiplozoon* Akhmerov, 1974 is paraphyletic and requires a taxonomic revision. The paraphyly of *Paradiplozoon* is a recognised scientific fact [13, 16, 23, 42, 45]. In the study that described *P. yarkandense*, the ITS-2 sequence grouped it with *Paradiplozoon bingolense* and *Paradiplozoon krugerense* [23]. This clade was recognised as “Genus 3” in a recent review of diplozoid phylogeny [13]. No other species nominally included in the “Genus 3” were available for our analysis, so *P. yarkandense* clustered with the only available “Genus 2” species—*P. opsariichthydis*. This is in agreement with the proposed sister group relationship of “Genus 2” (*Paradiplozoon*) and “Genus 3”, which together form the Clade2 [13]. Clade 1 comprised a number of different genera: *Eudiplozoon, Sindiplozoon, Inustiatus* and Genus 1. The Genus 1 clade comprised *Diplozoon* and some *Paradiplozoon* species, including the *P. homoion* complex [13]. *Sindiplozoon* sp. and *P. homoion* forming a separate clade in our analysis supports the above topology, as they correspond to the above described “Clade 1”. The authors proposed that the “Genus 1” clade should revert to the type genus *Diplozoon* von Nordmann, 1832 [13]. Following this proposal, which is also supported by our study, the species currently denominated *P. homoion* may have to be renamed *Diplozoon homoion*. Remarkably, “Genus 3” contains species from Africa, Turkey and an unidentified sequence from China [13]. Our findings provide an additional indirect indication that this may be a unique genus, sufficiently evolutionarily old to have such a wide distribution (alternatively the distribution may be of a recent origin due to global trade, but in that case, this should show as a poorly resolved topology). The authors also proposed that the appropriate designation for Genus 3 may be *Indodiplozoon*, given that all specimens were collected from river systems that eventually enter the Indian Ocean, which may indicate that members of this proposed genus have an Indo-Pacific ancestor [13]. Accordingly, *P. yarkandense* may have to be renamed *Indodiplozoon yarkandense*. This taxonomic revision is further supported by remarkably low identity values between genes of nominally congeneric *P. yarkandense* and *P. homoion*. Additionally, the geographic distribution of the genus *Indodiplozoon* indicates that it may be evolutionarily rather old. In this light, it should be noted that the species inhabits the endorheic Tarim Basin in Xinjiang, China. However, a number of rivers from Xinjiang and geographically close Tibet and Qinghai empty into the Indian Ocean, so this finding may not directly contradict the above hypothesis. We should note that the accuracy of taxonomic revision is affected by a limited number of morphological parameters useful for the identification and classification of these species, as well as insufficiently detailed previous morphological descriptions and unavailability of sufficiently detailed and complete morphological maps [12, 13]. As a result, currently we do not have morphological comparative data that perfectly support the molecular results, or we even have conflicting signals from molecular and morphological data. For example, molecular data indicate that *Paradiplozoon homoion* should be assigned to the genus *Diplozoon*, but morphologically this species better resembles the classical *Paradiplozoon* features: the anterior part of the body between the opisthaptor and the area of the reproductive organs does not have a prominent disc dilation [46, 47] (more details in Additional file 1: Text S1 and Figs. S1 to S5). Similarly, the three *Diplozoon* species that putatively belong to the Genus 3 (*Indodiplozoon*) also share the basic characteristics of the genus *Diplozoon* (a prominent disc dilation in the anterior part of the body) [47]. Therefore, in cases where morphological data do not directly support the molecular data, we should seek agreement between both mitochondrial DNA (mtDNA) and nuclear DNA (nucDNA) data before going forth with taxonomic revision.

Unfortunately, in some cases, mtDNA and nucDNA data sometimes also produce contradictory results. For example, *Eudiplozoon* forming a sister group to all other available Diplozoidae is in disagreement with the topology produced by the ITS-2 sequences, which indicates that more studies with new molecular data are needed to resolve the phylogeny of Diplozoidae. It may also be important to note that a study based on nuclear *18S* and *28S* genes found that Mazocraeidae was the only polyopisthocotylean family that exhibited an elevated evolutionary rate [9], but mitogenomic sequences do not follow this pattern: Diplozoidae exhibited the longest branches in the dataset. Although our results provide further indications that mitogenomes may be a useful tool in the phylogenetics of Diplozoidae and Polyopisthocotylea, there are currently too few mitogenomic sequences available to conclude this with confidence.

Our study provides further evidence that mitogenomes of Diplozoidae are evolving at elevated rates within the polyopisthocotylean dataset. This was reflected in their exceptionally long branches, large sizes, unique base composition, large base composition skews, and very low gene sequence similarity levels between the two newly sequenced species. As an example, even the normally highly conserved *cox1* gene [43] exhibited an identity value of only 71.67%, which is exceptionally low for metazoans on average, but not uncommon in Platyhelminthes [48]. Similar values were also observed in the previous Diplozoidae analysis [11]. Unique base composition and increased skews in Diplozoidae were also observed in the previous study [11]. Increased skews might be reflective of reduced purifying selection pressures [49], which would also explain long branches. The A+T-base content is commonly high in monogenean mitogenomes [2, 31, 32]. Remarkably, the elevated AT content in the Polyopisthocotylea is largely driven by the high T-base content, whereas the reduced AT content in Diplozoidae was driven solely by the strongly reduced A-content in comparison with other polyopisthocotylids. As their reduced A+T-base composition bias is reflective of their faster, and not slower, evolution, this indicates that the AT content of Diplozoidae might be evolving in the opposite direction of most other Monogenean mitogenomes, towards lower values. In some other aspects, the two newly sequenced mitogenomes possessed typical general characteristics of most flatworm and all neodermatan mitogenomes; e.g. all genes are transcribed from the same strand and the *atp8* gene could not be identified [8, 11, 50, 51]. It should be noted that we identified a number of putative ORFs in both large NCRs, but comparison with previously annotated flatworm atp8 genes did not reveal any similarity. Without gene and protein expression data, it remains impossible to assess whether these putative ORFs are expressed and functional. The gene order exhibited by *Eudiplozoon* sp. and *Sindiplozoon* sp. was previously recognised as the ancestral architecture for the Diplozoidae, and putatively even for Polyopisthocotylea [11]. Our study offers further support for this hypothesis, as both newly sequenced mitogenomes exhibit the putative ancestral gene order.

We identified remarkably large overlaps between genes. Usually, overlaps in metazoan mitogenomes involve tRNA genes, which is believed to be a consequence of lesser evolutionary constraints on tRNA sequences [44]. The overlaps between *atp6*/*atp8* and *nad4*/*nad4L* are common in a broad range of metazoan lineages, perhaps due to their evolutionary conserved translation from a bicistronic mRNA [19, 52–57] and the fact that *atp8* and *nad4L* are small genes that appear to evolve under relaxed evolutionary constraints, as evidenced by the absence of *atp8* from several major metazoan lineages [19]. Overlaps between PCGs of less than 10 bp have been experimentally confirmed in a nematode species [58], but the exceptionally large overlap between *cox1* and *nad4L* observed here appears to be very rare. Much larger overlaps involving tRNA genes have been observed and experimentally confirmed in *Armadillidium vulgare* (Arthropoda: Isopoda) [44]. However, this was explained by the apparent existence of strong evolutionary pressure for a reduced mitogenomic size [44], which does not appear to be the case in the newly sequenced Diplozoidae species, as they possess larger than average mitogenomes. While relaxed purifying selection pressures may also be a putative explanation for the existence of unusually large overlaps, their existence remains an evolutionary puzzle. Finally, although we did our best to confirm these overlaps by conducting detailed comparative analyses and considering alternative start and stop codons, without the transcriptomic data, we cannot be 100% confident that these are not annotation artefacts.

## Conclusions

Due to their comparative advantages, mitogenomic sequences are a popular marker for phylogenetic studies, but their applicability is still somewhat curbed by the fact that many taxonomic categories remain poorly or not at all represented. Furthermore, multiple studies have indicated that in some cases, mitogenomes may also produce artefactual relationships [59–61]. Although our initial findings are promising, future studies should be very cautious in this aspect, and seek agreement between the topology produced by mitogenomic, nuclear, and ideally morphological data, to assess whether mitogenomic data can be used with confidence to conduct a thorough revision of the taxonomy and phylogeny of Diplozoidae. Furthermore, as the reasons for their elevated evolutionary rates remain unknown, Diplozoidae are a remarkably interesting lineage for other types of evolutionary mitogenomic studies.

## Supplementary Information

**Additional file 1: Text S1.** Morphology of Diplozoidae. **Figure S1.** A drawing of a *P. homoion* specimen. **Figure S2.** *P. homoion*—anchor. **Figure S3.** *P. homoion*—clamp. **Figure S4.** *P. yarkandense*—holotype. **Figure S5.** Opisthaptor of *P. yarkandense*. **Figure S6.** BLAST results for the *ITS-2* of *P. yarkandense*. **Figure S7.** BLAST results for the *ITS-2* of *P. homoion*. **Figure S8**. *Nad6* 3′ elongation in P. homoion. **Figure S9**. Alignment of translated *nad4L* gene products of Polyopisthocotylea. **Figure S10.** Alignment of *trnI* genes of Polyopisthocotylea. **Figure S11.** Secondary structure of the *trnI* gene of *P. homoion*. **Figure S12.** Alignment of *trnG* genes. **Figure S13.** Alignment of *trnM* genes. **Table S1.** Primers used for sequencing and amplification of the complete mitogenome of *P. yarkandense.* **Table S2.** Primers used for sequencing and amplification of the complete mitogenome of *P. homoion.*

**Additional file 2: Dataset S1.** Taxonomic details and comparative mitogenomic data for the polyopisthocotylean mitogenomic dataset used. **Dataset S2.** Comparative gene data for the polyopisthocotylean mitogenomic dataset used.

## Data Availability

The two newly sequenced mitogenomes are available from GenBank under the accession numbers: OM525853 (*Paradiplozoon yarkandense*), and OM525852 (*Paradiplozoon homoion*). The *ITS-2* sequences are available under OM432149 (*Paradiplozoon yarkandense*), OM422697 (*Paradiplozoon homoion*). Other datasets supporting the conclusions of this article are included within the article and its additional files.

## Abbreviations

PCG: Protein-coding gene
bp: Base pair
