# Correction: The complete mitochondrial genomes of *Paradiplozoon yarkandense* and *Paradiplozoon homoion* confirm that Diplozoidae evolve at an elevated rate

**DOI:** 10.1186/s13071-022-05308-5

**Published:** 2022-05-16

**Authors:** Cui-Lan Hao, Kadirden Arken, Munira Kadir, Wen-Run Zhang, Meng-Jie Rong, Nian-Wen Wei, Yan-Jun Liu, Cheng Yue

**Affiliations:** College of Veterinary Medicine, Xinjiang Agricultural University, Urumqi, 830052 Xinjiang China

## Correction to: Parasites Vectors 15:149 (2022) https://doi.org/10.1186/s13071-022-05275-x

Following the original publication of this article [1], the authors flagged the following errors: Fig. 3 had been published upside-down; two newly sequenced species (Paradiplozoon yarkandense and Paradiplozoon homoion) had been mistakenly omitted from Figs. 4 and 5; and in the section ‘Gene overlaps’, in the sentence “A relatively large putative overlap between *cytb* and *nad4L* was conserved in both species: *P. yarkandense* = 10 bp, *P. homoion* = 16 bp (Table 1)”, *cox1* had been written instead of *cytb*.

**Fig. 3:**
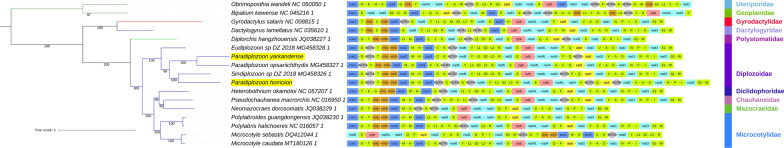
Gene orders in Polyopisthocotylea. Species names are followed by GenBank accession numbers. The two newly sequenced species are shaded yellow. Family-level taxonomic identity is shown to the right. Partial mitogenomes are labelled with the letter P

**Fig. 4:**
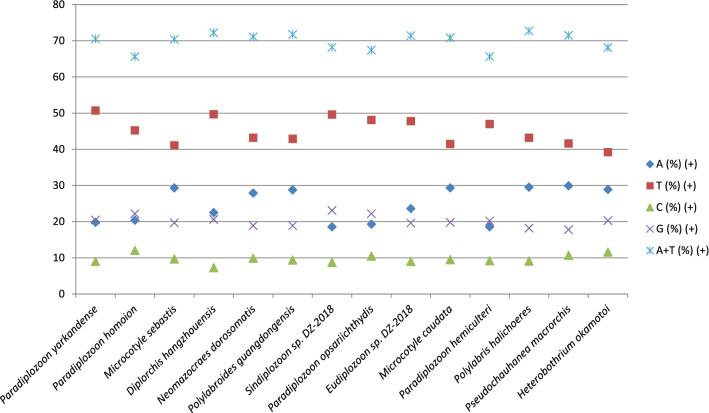
The base composition of mitogenomes of Polyopisthocotylea

**Fig. 5:**
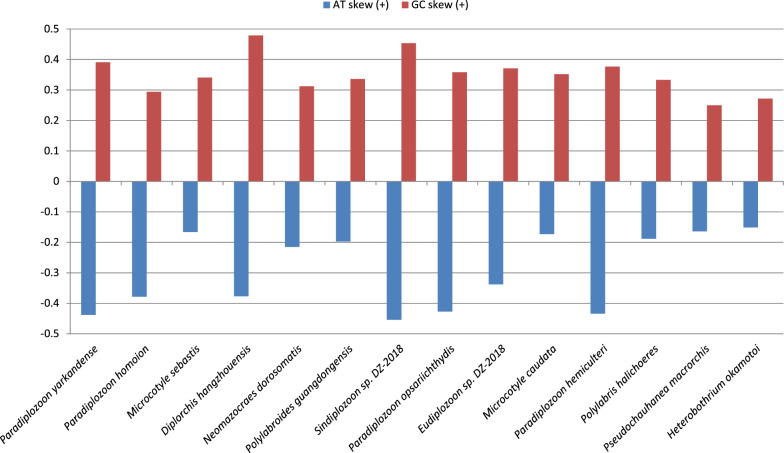
AT and GC skews in mitogenomes of Polyopisthocotylea

The original article has since been corrected and the corrected figures may be found in this erratum for reference.
