# Supplementary material for: The complete mitochondrial genomes of Paradiplozoon yarkandense and Paradiplozoon homoion confirm that Diplozoidae evolve at an elevated rate
Source: Parasit Vectors. 2022 Apr 27;15:149. doi: 10.1186/s13071-022-05275-x (PMC9044634; doi:10.1186/s13071-022-05275-x)
Supplement: Supplementary file 1 — Additional file 1: Text S1. Morphology of Diplozoidae. Figure S1. A drawing of a P. homoion specimen. Figure S2. P. homoion—anchor. Figure S3. P. homoion—clamp. Figure S4. P. yarkandense—holotype. Figure S5. Opisthaptor of P. yarkandense. Figure S6. BLAST results for the ITS-2 of P. yarkandense. Figure S7. BLAST results for the ITS-2 of P. homoion. Figure S8. Nad6 3′ elongation in P. homoion. Figure S9. Alignment of translated nad4L gene products of Polyopisthocotylea. Figure S10. Alignment of trnI genes of Polyopisthocotylea. Figure S11. Secondary structure of the trnI gene of P. homoion. Figure S12. Alignment of trnG genes. Figure S13. Alignment of trnM genes. Table S1. Primers used for sequencing and amplification of the complete mitogenome of P. yarkandense. Table S2. Primers used for sequencing and amplification of the complete mitogenome of P. homoion. [file 13071_2022_5275_MOESM1_ESM.pdf]

Additional file 1 for

The complete mitochondrial genomes of *Paradiplozoon yarkandense* and *Paradiplozoon homoion* confirm that Diplozoidae evolve at an elevated rate

Cui-Lan Hao, Kadirden Arken, Munira Kadir, Wen-Run Zhang, Meng-Jie Rong, Nian-Wen Wei, Yan-Jun Liu, Cheng Yue*

* Corresponding author (CY): yuechengxnd@aliyun.com

Text S1. Morphology of Diplozoidae

The morphology-based classification of the diplozoid genera discussed in this study is based on the morphology of the posterior region (they all have the same number of attachment clamps) [1]. *Paradiplozoon*: the anterior part of the body between the opisthaptor and the reproductive union area has no special disc dilation. *Diplozoon*: there are obvious and thick side folds before the disc dilation. *Sindiplozoon*: there is no special thick and big side fold before the disc dilation. *Eudiplozoon*: there is a pair of large, prominent round glands in front of the oral sucker area. *Inustiatus*: the genital foramen opens in the anterior middle side of the body.

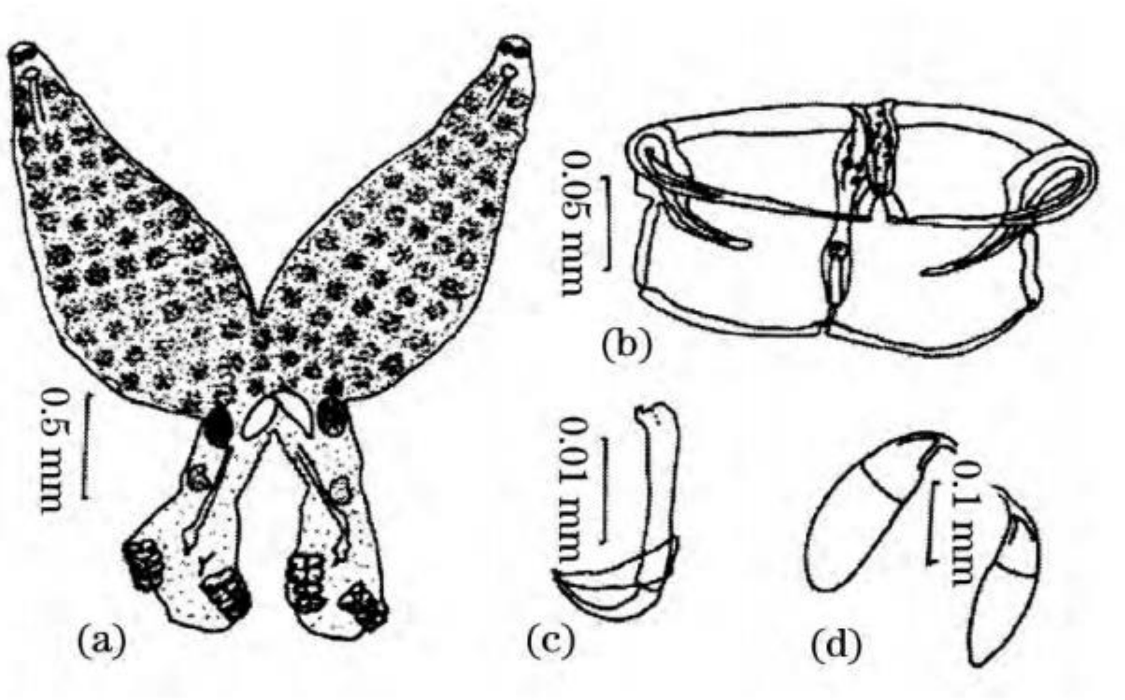

Figure S1. A drawing of a *P. homoion* specimen. (a) The ventral view of the adult; (b) the clamp; (c) the central hook; (d) the egg. The figure was taken from [2].

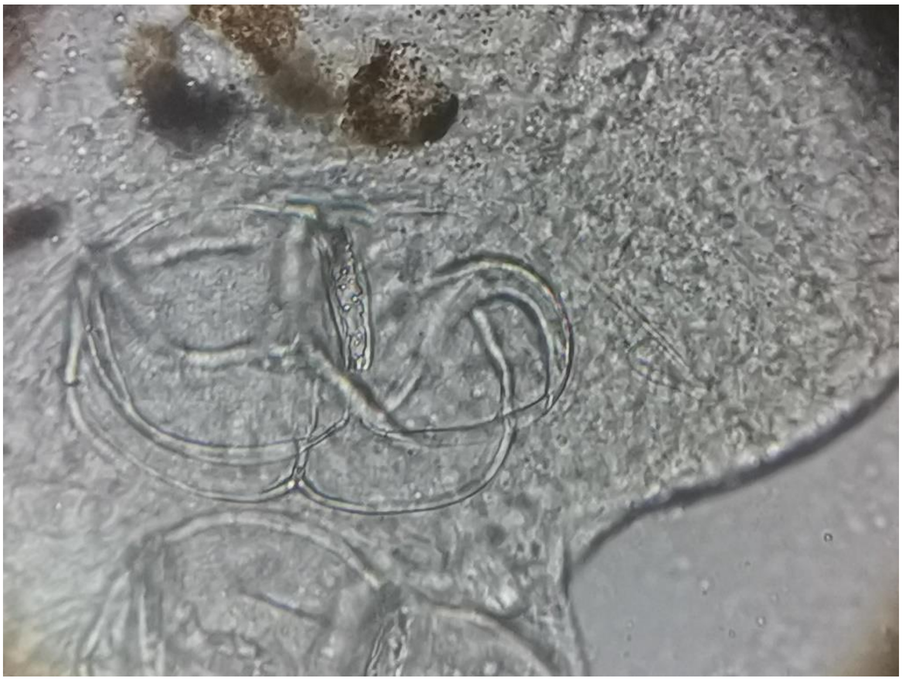

Figure S2. *P. homoion* – anchor. Magnification 10x.

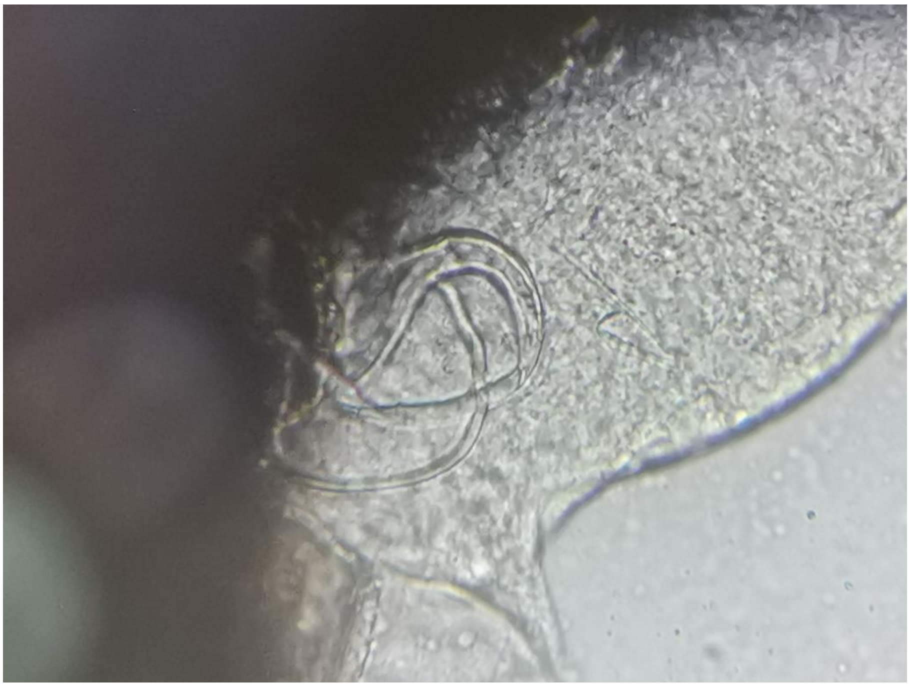

Figure S3. *P. homoion* – clamp. Magnification 10x.

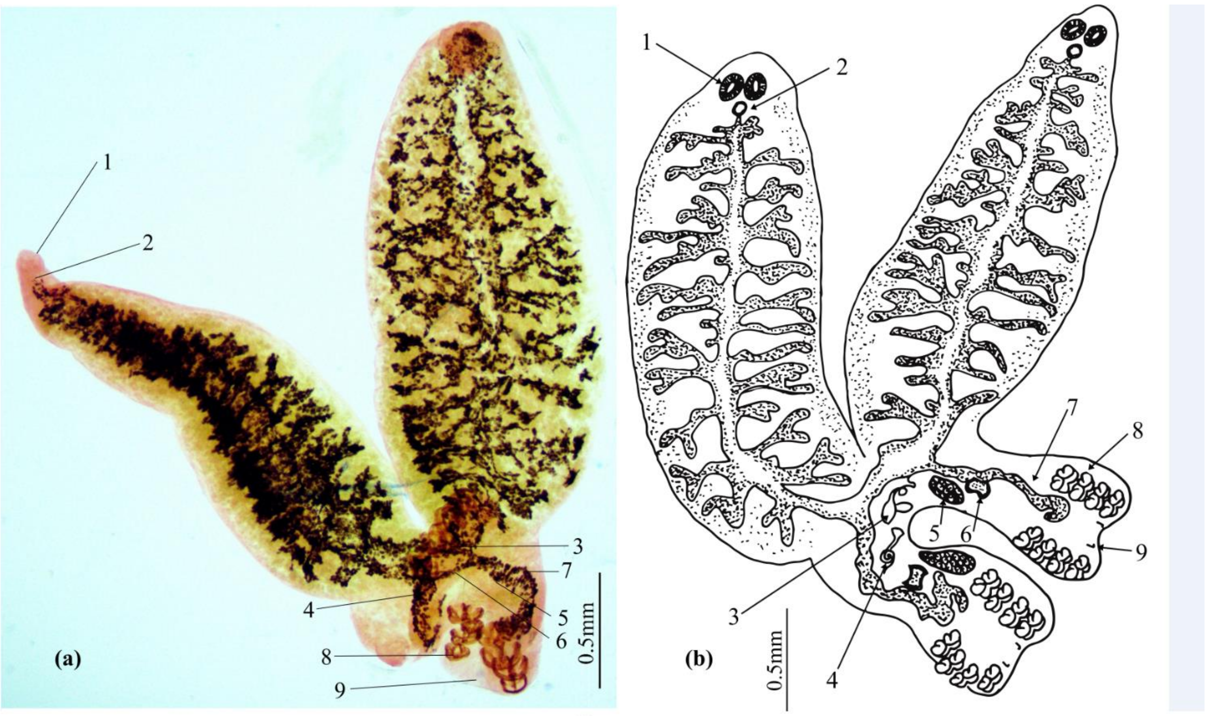

Figure S4. *P. yarkandense* – holotype. a) Ventral morphology of an adult fused pair of *P. yarkandense* spexcimens. b) Descriptive drawing of an adult fused pair. 1 – suckers, 2 - pharynx; 3 - eggs; 4 - egg filament; 5 - ovary; 6 - testis; 7 - anterior intestine; 8 - posterior intestine; 9 - clamps; 10 - anchors The figure was taken from [3].

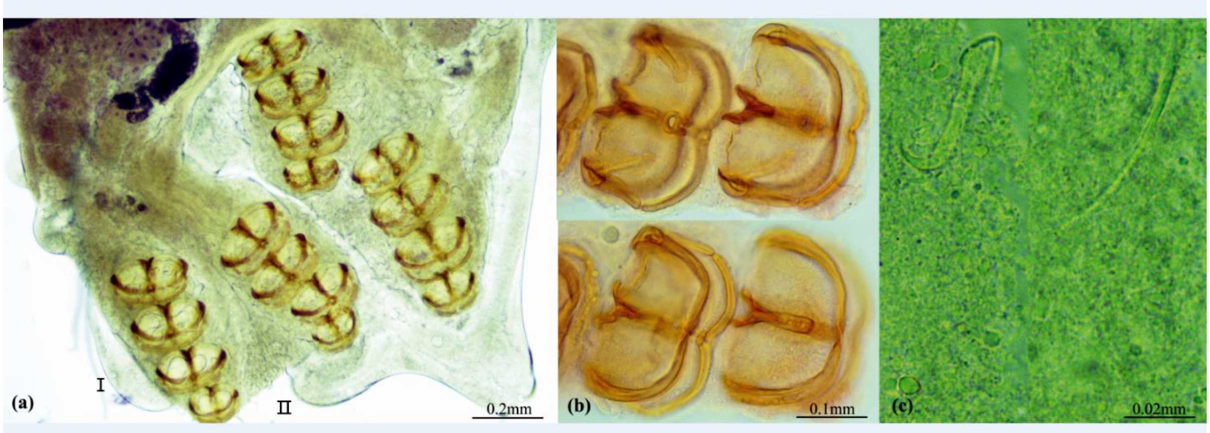

Figure S5. Opisthaptor of *P. yarkandense*. a) Four pairs of clamps on an opisthapor (bar, 0.2 mm). b) Clamps (arrow I in a), medial (black arrow) and lateral (white arrow) sclerite parts of a jaw (bar, 0.1 mm). c) Inward central hook (arrow II in a) (bar, 0.02 mm). The figure was taken from [3].

	Description	Scientific Name	Max Score	Total Score	Query Cover	E value	Per. Ident	Acc. Len	Accession
☑	Paradiplozoon sp. y KA-2020 isolate TX56 5.8S ribosomal RNA gene, partial sequence; internal transcribed space…	Paradiplozoon sp…	1384	1384	100%	0.0	100.00%	807	MN892639.1
☑	Paradiplozoon sp. y KA-2020 isolate TX43 5.8S ribosomal RNA gene, partial sequence; internal transcribed space…	Paradiplozoon sp…	1384	1384	100%	0.0	100.00%	807	MN892633.1
☑	Paradiplozoon sp. y KA-2020 isolate TX36 5.8S ribosomal RNA gene, partial sequence; internal transcribed space…	Paradiplozoon sp…	1384	1384	100%	0.0	100.00%	807	MN892638.1
☑	Paradiplozoon sp. y KA-2020 isolate TX81 5.8S ribosomal RNA gene, partial sequence; internal transcribed space…	Paradiplozoon sp…	1384	1384	100%	0.0	100.00%	807	MN892636.1
☑	Paradiplozoon sp. y KA-2020 isolate TX127 5.8S ribosomal RNA gene, partial sequence; internal transcribed spac…	Paradiplozoon sp…	1384	1384	100%	0.0	100.00%	807	MN892631.1
☑	Paradiplozoon sp. y KA-2020 isolate TX92 5.8S ribosomal RNA gene, partial sequence; internal transcribed space…	Paradiplozoon sp…	1384	1384	100%	0.0	100.00%	807	MN892635.1
☑	Paradiplozoon sp. y KA-2020 isolate TX103 5.8S ribosomal RNA gene, partial sequence; internal transcribed spac…	Paradiplozoon sp…	1384	1384	100%	0.0	100.00%	807	MN892634.1
☑	Paradiplozoon sp. y KA-2020 isolate TX111 5.8S ribosomal RNA gene, partial sequence; internal transcribed spac…	Paradiplozoon sp…	1384	1384	100%	0.0	100.00%	807	MN892632.1
☑	Paradiplozoon sp. y KA-2020 isolate TX22 5.8S ribosomal RNA gene, partial sequence; internal transcribed space…	Paradiplozoon sp…	1384	1384	100%	0.0	100.00%	807	MN892630.1
☑	Paradiplozoon sp. y KA-2020 isolate TX12 5.8S ribosomal RNA gene, partial sequence; internal transcribed space…	Paradiplozoon sp…	1376	1376	100%	0.0	99.87%	806	MN892637.1
☑	Paradiplozoon bingolensis genomic DNA containing 5.8S rRNA gene, ITS2 and 28S rRNA gene	Paradiplozoon bi…	1002	1002	94%	0.0	92.13%	725	HE653910.1

Figure S6. BLAST results for the *ITS-2* of *P. yarkandense*.

	Description	Scientific Name	Max Score	Total Score	Query Cover	E value	Per. Ident	Acc. Len	Accession
☑	Paradiplozoon homoion voucher Paradiplozoon_homoion_Spain_BAHA_KV_ITS2 internal transcribed spacer 2,...	Paradiplozoon h...	1186	1186	87%	0.0	100.00%	642	MT417728.1
☑	Paradiplozoon homoion isolate A1 5.8S ribosomal RNA gene, partial sequence; internal transcribed spacer 2, co...	Paradiplozoon h...	1349	1349	100%	0.0	99.86%	820	MT028131.1
☑	Paradiplozoon skrjabini 5.8S ribosomal RNA gene, partial sequence; internal transcribed spacer 2, complete seq...	Paradiplozoon s...	1349	1349	100%	0.0	99.86%	764	KP340974.1
☑	Paradiplozoon gracile 5.8S ribosomal RNA gene, partial sequence; internal transcribed spacer 2, complete sequ...	Paradiplozoon gr...	1349	1349	100%	0.0	99.86%	764	KP340973.1
☑	Paradiplozoon homoion 5.8S ribosomal RNA gene, partial sequence; internal transcribed spacer 2, complete se...	Paradiplozoon h...	1349	1349	100%	0.0	99.86%	764	KP340972.1
☑	Paradiplozoon homoion partial 5.8S rRNA gene, ITS2, and partial 28S rRNA gene	Paradiplozoon h...	1349	1349	100%	0.0	99.86%	780	AJ300715.2
☑	Diplozoon homoion 5.8S ribosomal RNA gene, partial sequence; internal transcribed spacer 2, complete sequen...	Paradiplozoon h...	1310	1310	97%	0.0	99.86%	996	AF369760.1
☑	Paradiplozoon homoion voucher 5A 5.8S ribosomal RNA gene, partial sequence; internal transcribed spacer 2,...	Paradiplozoon h...	1271	1271	94%	0.0	99.86%	691	MW309386.1
☑	Paradiplozoon homoion voucher Paradiplozoon_homoion_Greece_TEBE_KV_ITS2 internal transcribed spacer ...	Paradiplozoon h...	1181	1181	87%	0.0	99.84%	642	MT417729.1
☑	Paradiplozoon skrjabini genes for 18S rRNA, ITS1, 5.8S rRNA, ITS2, 28S rRNA, partial and complete sequence,...	Paradiplozoon s...	1151	1151	91%	0.0	97.91%	683	LC050528.1

Figure S7. BLAST results for the *ITS-2* of *P. homoion*.

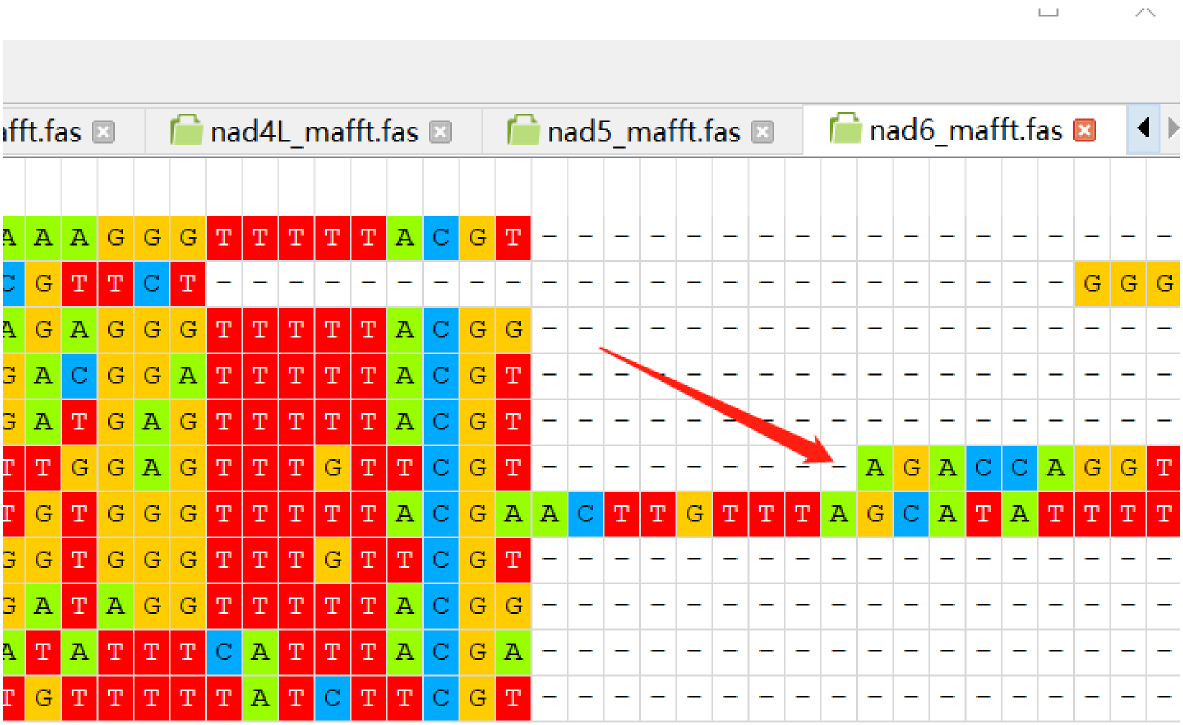

Figure S8. *Nad6* 3’ elongation in *P. homoion*. It is marked by a red arrow.

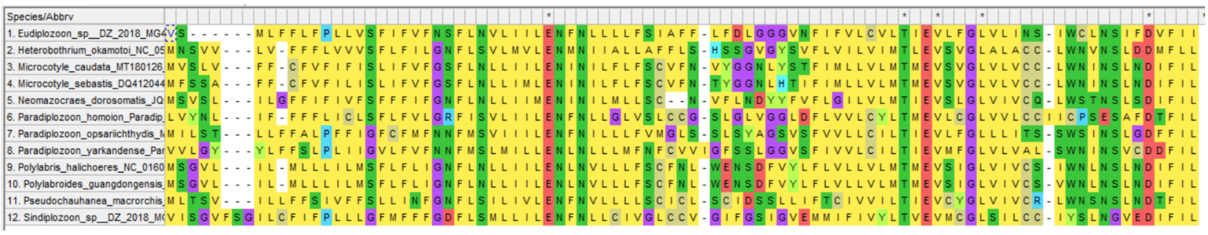

Figure S9. Alignment of translated *nad4L* gene products of Polyopisthocotylea.

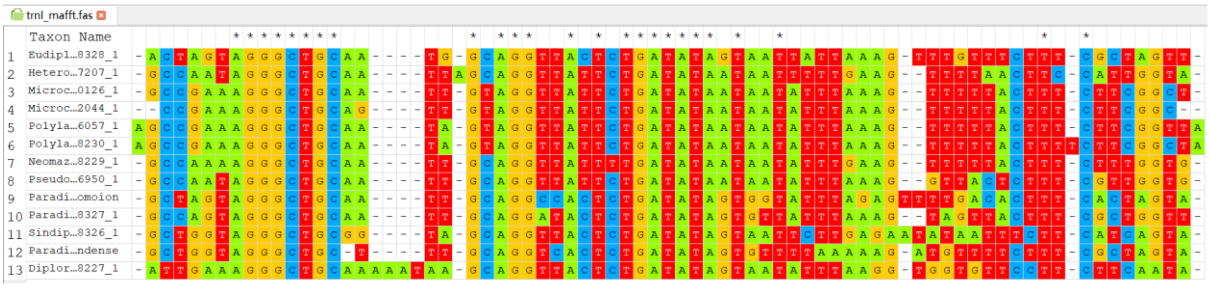

Figure S10. Alignment of *trnI* genes of Polyopisthocotylea. Paradi…ndense is *Paradiplozoon yarkandense* and Paradi…omoion is *Paradiplozoon homoion.*

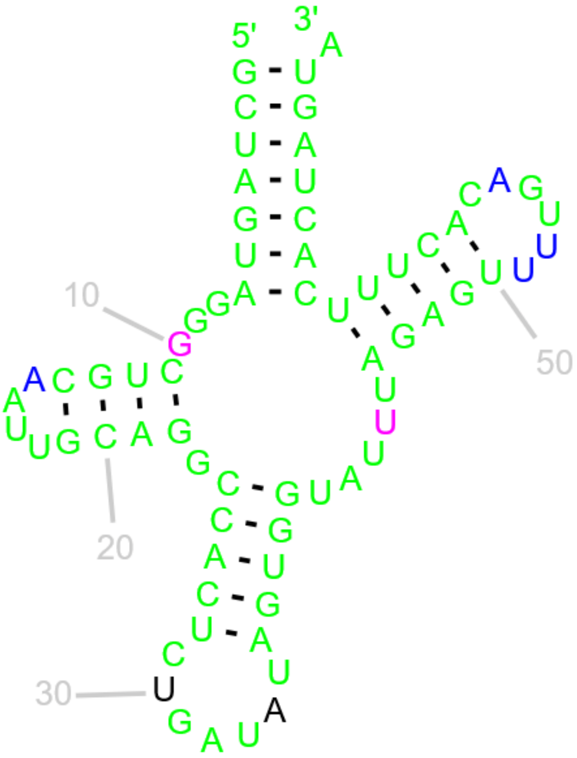

Figure S11. Secondary structure of the *trnI* gene of *P. homoion*.

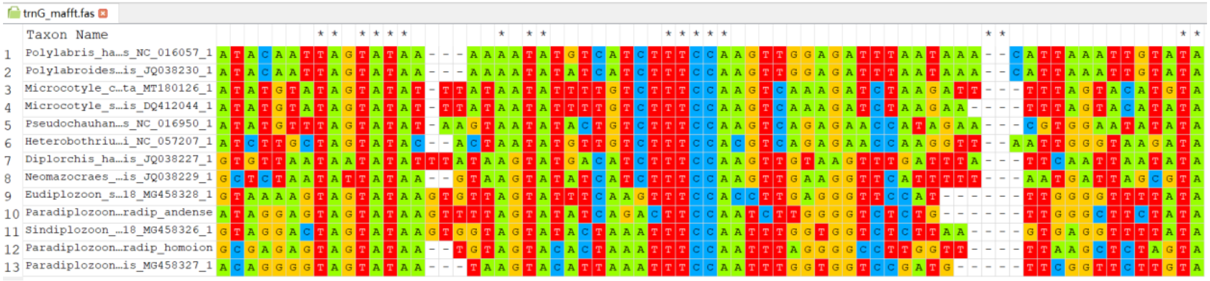

Figure S12. Alignment of *trnG* genes. Paradiplozoon…radip_ andense is *Paradiplozoon yarkandense*. Paradiplozoon…radip_ homoion is *Paradiplozoon homoion.*

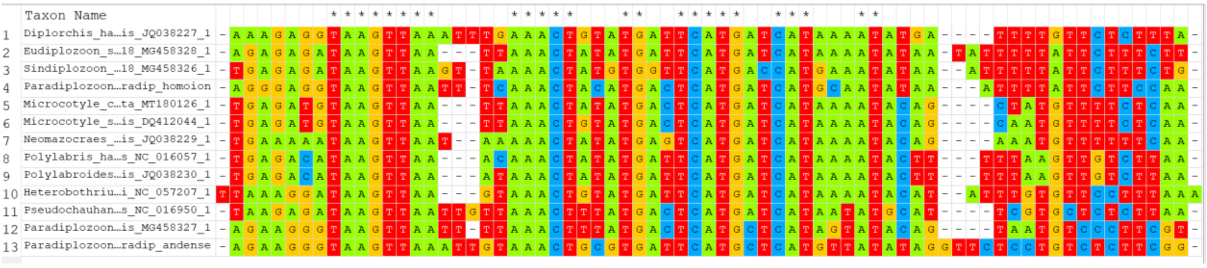

Figure S13. Alignment of *trnM* genes. Paradiplozoon…radip_ andense is *Paradiplozoon yarkandense.* Paradiplozoon…radip_ homoion is *Paradiplozoon homoion.*

Table S1. Primers used for sequencing and amplification of the complete mitogenome of *Paradiplozoon yarkandense.*

**Fragment No.**	**Gene or region**	**Primer name**	**Sequence (5'-3')**	**Length (bp)**
F1	ND5	T1F1	GAGGCTATGCGTGCTCCTAC	218
		T1R1	CTAGTAGACAAAGCAACAATC	
F2	ND5-CYTB	T1F2	TGGTGTTGTAGGTTTGGATG	1533
		T1R2	CCAAAACTAACATGAACAC	
F3	CYTB	T1F3	TCAGATGTCTTATTGAGCTG	451
		T1R3	CTCCACACAACGTAAAGTAGC	
F4	CYTB-ND4	T1F4	GTCAAGTTGATTTCCTTATTTAAC	1164
		T1R4	CAAACCTACCCAACAAGGGT	
F5	ND4	T1F5	GATTCTCCTTATTCTGATCG	242
		T1R5	GCCTCAGCATGAACAACAGG	
F6	ND4-ND1	T1F6	TTGAGGGTTTGTTGATGAC	3523
		T1R6	GTAAGAAACACAAACTCTAGC	
F7	ND1	T1F7	GTTTGAACGTAAGTTGTTGG	549
		T1R7	GAATAGTCAAAAGGACTACG	
F8	ND1-COX1	T1F8	GTTTGATGTTGGTTTTTGGTG	1472
		T1R8	GTACCAACACCTCTATAAAGC	
F9	COX1	T1F9	GGATGATATGAAGCTTCCG	1086
		T1R9	GAATCATAAGAACAAACACG	

F10	COX1-16S	T1F10	GCTGTGTTATGGTGGTGACC	1489
		T1R10	GTAGAATCTGTACTACTAGC	
F11	16S	T1F11	TAGCCGCATTAGCCTGAGTG	410
		T1R11	GATTTACATCGGTCTTAACTC	
F12	16S-12S	T1F12	GCTACCTCGATGTTGATTTAAG	501
		T1R12	CAATAAGAGGGTCTCTAATCC	
F13	12S	T1F13	CTGATCGCTCTTTTAACTCC	502
		T1R13	CAACTAAGGTCGAAATTGACG	
F14	12S-COX2	T1F14	GTAACAATTTTGTAGGTAGG	543
		T1R14	AACCTAGAACTAACTCCATC	
F15	COX2	T1F15	GGTCATCAGTGATACTGAGAG	276
		T1R15	AGCGCCACAAAACTCGCTAC	
F16	COX2-ND5	T1F16	GATGCTGTTCCTGGTCGAG	4829
		T1R16	CACCAACACCATACTCTAAC	

Table S2. Primers used for sequencing and amplification of the complete mitogenome of *Paradiplozoon homoion.*

**Fragment No.**	**Gene or region**	**Primer name**	**Sequence (5'-3')**	**Length (bp)**
F1	ND5	BJF1	GAGGCCATGCGTGCTCCTAC	212
		BJR1	GTTGACATGGCCACCCCCTTC	
F2	ND5-CYTB	BJF2	ATTCTTTTGTCTTGTCTAGG	1544
		BJR2	CACCAAACCTAACATGTAAGC	
F3	CYTB	BJF3	CAGATGTCTTATTGAGCTGG	538
		BJR3	AGCCACAGGGGGAGCTCCAAAT	
F4	CYTB-ND1	BJF4	GCCTATCCTTGAATGACTAG	4333
		BJR4	GATCCAAACCAAGAACCAAG	
F5	ND1	BJF5	TAGAGCGAAAGGTTTTGGG	577
		BJR5	CCCCTAACAAAATCTCTCTC	

F6	ND1-ND3	BJF6	AGTTGGCTTTCTTCCTTTGG	707
		BJR6	CAATCGAGCAATTGAGACAC	
F7	tRNA-Ile-16S	BJF7	TTGCAGGCCACTCTGATATAG	2413
		BJR7	CCGATGATCATAATGCAAAAGG	
F8	COX1-12S	BJF8	CCAACAGTTGCTAGTGTTG	1586
		BJR8	GTCACTAATCCCTGTCTCTA	
F9	16S-COX2	BJF9	CTCGATGTTGACTTAAGAG	1381
		BJR9	GGAATGAAGAACATCAGTAG	
F10	COX2	BJF10	ATAGGTCGTCAGTGGAATTG	284
		BJR10	GCTCCACAAAACTCACTACA	
F11	COX2-ND5	BJF11	TGAAGATTGATGCCGTACC	5255
		BJR11	GAACTGACAAAACCTAAACC	
F12	tRNA-Leu-ND5	BJF12	ATGGGCTAGTTTTAAGCGC	840
		BJR12	AAGCTAGTAAGACAAGTAGC	

References

1. Galli P, Pugachev O, Kritsky D. Guide to Monogenoidea of freshwater fish of Palaearctic and Amur regions. Ledizioni; 2010;

2. Xin W, Li J, Jia S, Wang N, Hao C, Zhu M, et al. A New Record of Diplozoidae in China. Arid Zone Research. 2014;31:1121–4.

3. Arken K, Hao C-L, Guo A-M, Zhang W-R, Rong M-J, Kamal W, et al. A New Species of Paradiplozoon (Monogenea: Diplozoidae), A Gill Parasite of the Schizothorax Fish (Cyprinidae: Schizothoracinae) from the Yarkand River, Xinjiang, China. Acta Parasit [Internet]. 2021 [cited 2021 Dec 1]; Available from: https://doi.org/10.1007/s11686-021-00466-5
